# The Role of Wait Time During the Questioning of Children: A Systematic Review

**DOI:** 10.1177/15248380241246793

**Published:** 2024-04-25

**Authors:** Annie Yun An Shiau, Kelly McWilliams, Shanna Williams

**Affiliations:** 1 McGill University, Montreal, QC, Canada; 2 John Jay College of Criminal Justice and the Graduate Center, CUNY, New York, NY, USA

**Keywords:** forensic interview, wait time, childhood and adolescents, interviewing practices, pauses

## Abstract

The benefits of wait time in classroom discourses have been well documented in the field of education since the 1970s. While current forensic interview guidelines recognize the importance of pauses, whether there is sufficient empirical evidence to inform wait time guidelines in the legal context remains unanswered. This systematic review aimed to synthesize and provide a holistic update on the available research on the role of wait time when questioning children and recommended future direction to develop wait time guidelines specific to child forensic interviews. Systematic searches were conducted using four databases (PsycINFO, MedLine, ERIC, and Scopus). A total of 3,953 unique articles were returned, following a title and abstract screening, 68 full texts were reviewed, and 26 (including five additional studies identified through a hand search) were included. Inclusion criteria were the study sample included children under 18, published a measure of wait time in a questioning context, and in English. Overall, most knowledge of wait time remains in the field of education. Natural wait time is short, but with training, extended wait time yields significant benefits for both child and adult talk. Only one study examined the role of wait time in the forensic interviewing setting where a 10-s wait time appears to be more productive than shorter pauses. Extended wait time is a promising and simple interviewing practice with the potential to facilitate children’s disclosure. The current review is a call for research in the area as it pertains to forensic interviewing of children and youth.

Child witnesses play an important role in cases of maltreatment, as they are often the only sources of information (e.g., Cross & Whitcomb, 2017). However, child witnesses tend to be more reluctant (Lyon et al., 2020), more susceptible to suggestion (Leichtman & Ceci, 1995), and more vulnerable to misinformation (Ceci & Bruck, 1995) compared to adults. Despite this, substantial research has shown that children can remember details of incidents and can act as reliable witnesses (McWilliams et al., 2012). To improve the quality of children’s reports, standardized interviewing protocols (e.g., National Institute of Child Health and Human Development Investigative Interview Protocol) are used to facilitate their disclosures (Lamb et al., 2018; Orbach et al., 2000). These protocols emphasize open-ended questioning, the use of ground rules, and non-suggestive support within interviews, all of which improve the quality and quantity of information in children’s reports (Lyon et al., 2019). While substantial gains have been made in forensic interviewing (Bruer et al., 2022), questions remain regarding support for children during the use of these protocols (McWilliams et al., 2021; Stolzenberg et al., 2021), particularly reluctant children (Gongola et al., 2021).

One area of support that has been included within these protocols is wait time. The use of open-ended questions can be cognitively demanding as they often require children’s recall, as opposed to recognition, of information (Williams et al., 2020). Because of this, it is important to give children time to respond. Currently, protocols provide guidelines regarding wait time; however, the research on wait time within the context of forensic interviews is not as robust as one may assume. Therefore, a review of wait time within interviews with children is needed.

## Defining Wait Time

Pauses are common in our everyday conversations and are important to allow time for both the speaker and listener to cognitively process information. However, natural wait times in conversations are brief. For adults, a pause under 200 ms between one speaker’s question and the other speaker’s response remains fairly constant across languages and cultures (Stivers et al., 2009). Children, on the other hand, tend to wait longer (mode of 300–400 ms) than adults when answering a question (Stivers et al., 2018). However, both children and adults tend to pause longer when they do not know the answer to a question or when they disagree with the questioner (Stivers et al., 2018). Questioners often find extended wait time awkward and respond by quickly rephrasing the question or providing additional information to clarify the question, instead of waiting, through the silence, for an answer (Macbeth, 2004; McHoul, 1990).

Although wait time is present and important for both forensic interviewing and classrooms, our current knowledge of wait time largely stems from research in education, where it has been studied for decades (e.g., Rowe, 1974). Rowe (1974), one of the early pioneers of wait time research, identified two types of wait time: (a) the pause after a teacher solicitation and before a student response or another teacher utterance (Wait Time 1) and (b) the pause after a student response and before a teacher utterance (Wait Time 2). Similar pauses may be observed during forensic interviews. For example, the time interviewers remain silent for children to recall and describe a past event (Rowe’s (1974) Wait Time 1) and the pause after children’s responses to formulate a follow-up question or to provide space for continual recall after the initial burst (Rowe’s (1974) Wait Time 2). Since Rowe’s (1974) pioneering work, some researchers have modified the term (e.g., “think time,” “student/teacher talk”; Gambrell, 1983; Jegede & Olajide, 1995) and the definition for wait time to be examined empirically. Most noteworthy, Tobin (1980) redefined wait time as any pause preceding a teacher utterance to address the influence of student talk on teacher wait time. This definition allowed for greater ease in manipulating wait time, as it was only necessary for teachers to extend their pause before commencing to speak, while students do not affect wait time duration.

A review conducted during the early growth of wait time research found that extending pauses in classrooms was associated with positive changes in teacher (e.g., less teacher talk, increase in the level of cognitive questions) and student talk (e.g., longer responses, less failure to respond; K.Tobin, 1987). In their critical analysis of the literature, Ingram and Elliott (2016) found the same results but argued for a more nuanced understanding of this tool, such that the context and desired outcomes of the conversation must be taken into consideration before determining the optimal wait time. Consistent with this point, we must use caution when extracting findings from the education literature to guide wait times in forensic interviews. It is important to acknowledge that wait time research predates the conception of forensic interviewing. While research in forensic interviewing has grown in recent years, the empirical attention toward wait time has decreased in the early 2000s. It is possible that the dynamics and goals of interviewing call for wait times that vary from those in educational settings. Although existing knowledge on wait time shows potential in its application to forensic interviewing, no research has directly examined it in forensic interviewing with children and no systematic review synthesizing the findings on wait time and its implications for forensic interviewing has been conducted.

## Wait Time in Forensic Interviews

### Benefits of Wait Time in Forensic Interviews

Similar to the need for teachers to provide learners sufficient cognitive processing time ( K.Tobin, 1987), forensic interviewers must also allow time for children to respond given the cognitive demands of forensic interviews. For example, during forensic interviews, children are often asked open-ended prompts (e.g., “tell me everything that happened”). Previous research has found that when faced with cognitively demanding questions, adults need significantly more time than our natural pauses (i.e., under 1 s). Ellsworth et al. (1991) found that college students require up to 32 s when answering questions involving higher-order thinking, including open-ended questions. Provided that children usually pause longer than adults in daily conversations (Stivers et al., 2018), child witnesses would likely require even longer time to answer open-ended prompts. In addition, certain child witnesses face additional barriers, such as non-native English speakers and children with disabilities. Some interviewing protocols acknowledge these barriers and recommend interviewers slow down, ensure clear pronunciation, and modify the questions asked (e.g., ask shorter and more directive, option-posing questions; Lamb et al., 2018). However, along with these accommodations, it may also be important that children are given enough time to answer before simplifying and turning toward direct or option-posing questions, which often elicit less detailed responses that are more prone to errors (Williams et al., 2020).

### Risks of Wait Time in Interviews

Although some children may simply need more time to answer, one must be cautious about giving child witnesses unlimited time during forensic interviews. It has been well documented that many suspected victims of abuse are reluctant to disclose (Lyon et al., 2020), whether to protect a familiar perpetrator (Paine & Hansen, 2002) or because of feelings of shame (Saywitz et al., 1991) or fear (Paine & Hansen, 2002). When examining forensic interviews with reluctant or non-disclosing children, researchers have found a stark difference in response and interview styles. Children who are reluctant to disclose are more likely to provide uninformative responses, especially omissions (e.g., remaining silent, saying “I don’t know”), even during the earliest, rapport-building phase of the interview (Hershkowitz et al., 2006). When asked about details of their allegations, reluctant children are more likely to use silence as a strategy to defer responding compared to explicit statements of reluctance (e.g., denials, “I don’t want to answer that”) and digressions (e.g., “want to see my shoes?”; Ahern et al., 2019). In response, interviewers build a more fragile rapport, pose more suggestive and option-posing questions, and provide fewer supportive comments (e.g., Ahern et al., 2019; Hershkowitz et al., 2006; Nogalska et al., 2022). While using sufficient wait time when interviewing a reluctant child is important, providing extensive wait time may be counter-effective. Reluctant witnesses could interpret long wait times as the interviewers’ tolerance to their deferral strategy or cause the interview to drag on inefficiently.

### How Much Time Is Enough Time?

Current forensic interview protocols acknowledge the importance of pauses and encourage interviewers to give children sufficient time to answer (Newlin, 2015). However, while some recommend interviewers to “wait for a response” following a question (Lamb et al., 2018), the exact duration is unclear. A limited number of studies in forensic interviewing have reported the use of wait times in their experimental design, where researchers have defaulted to a 10-s pause (e.g., Poole & Dickinson, 2011). However, wait time has not been an experimental variable examined, and whether, or how wait time affected the outcomes of interviews with children was not reported. Nonetheless, it remains uncertain whether this guideline has been adequately tested and whether the effects of wait time observed in classrooms can be generalized to forensic interviews with children. Wait time plays an important role in multiple processes through a child witness’ legal journey. There is a dire need to understand and inform guidelines for the use of pauses for children in the legal setting.

## The Current Study

The current study systematically reviewed the empirical literature on the practice and role of wait time when questioning children and adolescents across a large range of development. By analyzing literature across the various fields that examine this practice (e.g., education, speech sciences, psychology), this review aimed to provide an update on and critically evaluate the current knowledge of (a) adults’ natural wait time when questioning children and adolescents, (b) the effectiveness of various wait time training, (c) the outcome of various experimentally manipulated wait time, (d) adults’ natural wait time across question type, and (e) other factors when considering adult wait time when questioning children and adolescents. This review described the implications of forensic interviewing with children and identified future directions required to inform best-practice wait time guidelines for forensic interviews with children.

## Method

### Search Strategy

This review adhered to the Preferred reporting items for systematic reviews and meta-analyses (PRISMA) statement (Page et al., 2021) in designing a review protocol. Systematic searches were conducted using four databases: PsycInfo, MedLine, ERIC, and Scopus, for studies published between January 1, 1974, to the date of the initial search, May 16, 2022. In collaboration with a social sciences librarian, a search strategy was developed for each database: (Interview* OR question* OR interrog* OR “teacher student interact*”) AND (Silence OR prompt* OR “speech pause*” OR “wait time” OR “wait-time” OR “pause time” OR “pause-time”) AND (Infant OR child* OR teen* OR adolesc* OR “young adult”). An updated search including keywords more specific to the forensic context (i.e., Forensic Psychology, Forensic Interview) was conducted in November 2023.

### Eligibility Criteria

The following criteria were used to select studies to be included in the final analysis: (a) sample included children and adolescents (0–18 years), (b) included a measure of wait time/pause post-adult solicitation and/or post-child response in an interviewing or questioning context, either utilizing a well-established definition (e.g., Rowe, 1974 or Tobin, 1980) or a definition close in resemblance. As the review would like to examine original data that quantified wait time, studies were excluded based on the following criteria: (a) reviews, (b) books, (c) conference abstracts, (d) doctoral theses, (e) full text not available in English, and (f) abstract only. Studies were not excluded based on training methods or analytic techniques.

### Selection Process/Screening Protocol

**Figure 1.**
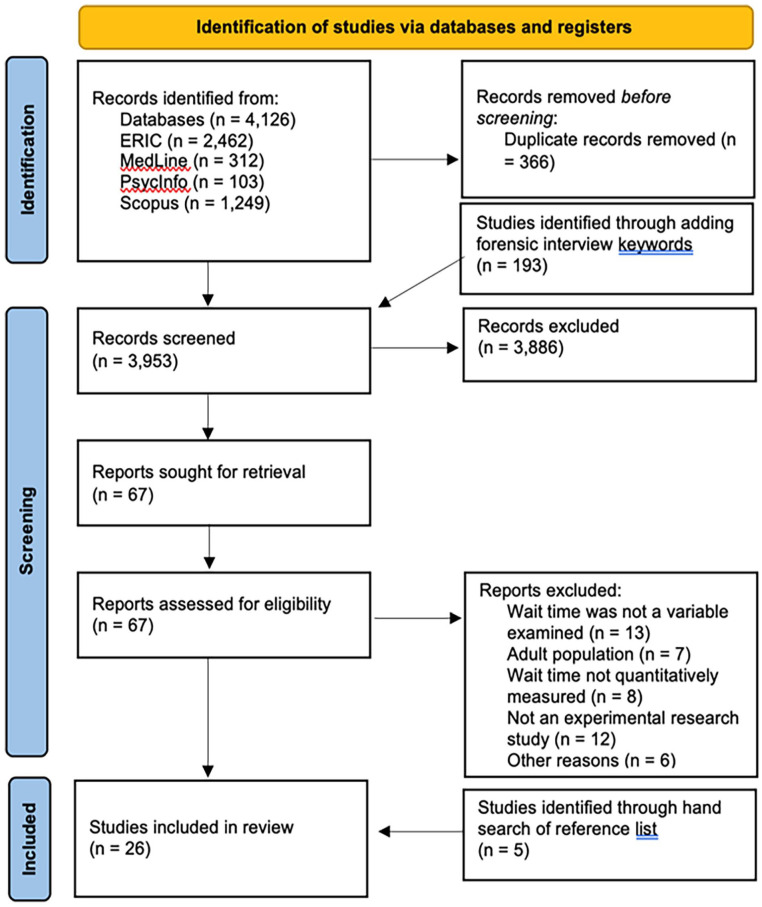
PRISMA flowchart. *Note*. PRISMA = preferred reporting items for systematic reviews and meta-analyses.

The PRISMA flow chart depicted in Figure 1 illustrates the process of literature search and selection. Our search returned 4,126 articles. A total of 3,760 unique articles remained after duplicates were removed. Using the prespecified eligibility criteria to answer the research questions regarding the current knowledge of adult wait time when interviewing children, four reviewers screened the title and abstract of each article for potential eligibility. In all, 68 articles were identified as potentially relevant. Three independent reviewers then screened the full text of the 68 articles using the eligibility criteria. Of those, 21 met the full eligibility criteria. A hand search of the reference lists of included articles was conducted, and five additional studies were identified. An updated search including keywords regarding the forensic context was conducted, and an additional 193 unique articles were identified. Upon screening the abstract and title, no articles fit the full eligibility criteria. Thus, a total of 26 articles were included in the final review. Inter-rater reliability between the first two authors was assessed using Cohen’s *K*. The inclusion decision for each article was dichotomously coded as either “include” or “exclude” based on the predetermined criteria. The *K* statistic for the article inclusion was found to be .835 (95% CI [0.649, 0.944], *p* < .001), indicating a strong agreement between the two raters.

### Quality Assessment

The STROBE checklist was used to assess the quality of the included studies (von Elm et al., 2007). The STROBE statement uses a 22-item checklist, and two researchers (AYAS and SW) performed the quality assessment independently, and the two results were compared. If differences are found, the two researchers discussed their ratings, and when needed, reread the articles. The third researcher (KM) was consulted for further disputes.

### Data Abstraction

An in-depth examination of the 26 articles meeting full eligibility criteria was conducted. A data abstraction table, specific to the aims of the review, was developed. The table included abstraction domains including study design, sample characteristics, definition of wait time, measures of wait time, training methods, analytic methods, key results, and study strengths and limitations. The first author abstracted data from each article and the third author confirmed the abstracted information. Discrepancies were resolved through discussion and consensus.

## Results

Results are synthesized and organized according to measures of wait time.

### Demographics: Setting, Age, and Characteristics of Students

Of the 26 articles included in the review, 23 were experimental studies in classrooms, 2 were experimental studies in research laboratories, and 1 was an observational study within children’s residences. In the 23 classroom studies and the observational study in the home, the children were interviewed by teachers. In the remaining two studies, children were interviewed by researchers or their mothers. The age of children being interviewed varied, four included preschool samples (under grade 1), six sampled elementary students (grades 1–6), six sampled middle school students (grades 6–8), and six sampled high school students (grades 9–12). Two included elementary and middle school students, one sampled elementary and preschool students, and one sampled middle and high school students. Most children sampled were typically developing, with three studies including atypically developing children (i.e., Down syndrome, cerebral palsy, and other unspecified special needs). In all, 13 studies were conducted in the United States, three in Australia, and eight studies in various other countries (i.e., Brunei, Germany, Nigeria, Portugal, Thailand, and Turkey). Wait time was measured through video recording (*n* = 6), audio recording (*n* = 15), an observation made in-person with paper and pencil (*n* = 2), or servo-chart recorders (i.e., a device that graphically records voice patterns; *n* = 1). Two studies did not specify how wait time was recorded or measured.

### Defining and Measuring Wait Time

Various definitions and measurements of wait time emerged. In all, 14 studies in the present review adopted Rowe’s (1974) definition and measurement of wait time. An additional two studies incorporated a similar conceptual distinction under alternative terminologies (i.e., think time, student/teacher talk; Gambrell, 1983; Jegede & Olajide, 1995). However, multiple conversational patterns could emerge from Rowe’s (1974) Wait Time 1 and 2. Three studies included in the current review measured Rowe’s wait times (1974) in more depth. Five studies, four of which were co-authored by Tobin, adopted Tobin’s (1980) definition of wait time (Tobin, 1980; K.Tobin, 1984, 1986; K. G.Tobin & Capie, 1982; Vettel & Windsor, 1997). One study (Baysen & Baysen, 2010) utilized a definition closely aligned with Tobin’s (1980).

One article deviated from the above categories. In contrast to examining the pause after a teacher question, Lee et al.’s (1987) measurement of wait time began after the presentation of verbal stimuli and terminated when the teacher posed a related question. Overall, despite slight variations in the definitions and measurements of wait time, all studies reviewed shared the common objective of assessing the time children were given before responding and the time teachers allowed themselves before resuming the conversation. Provided the variations, results will be discussed separately for Wait Time 1, Wait Time 2 (Rowe, 1974), and Wait Time General (Tobin, 1980). See Table 1 for details of wait time definitions and Supplemental Appendix A for a visual representation of the three most common measurements of wait time.

**Table 1.** Definitions of Wait Time.

Studies	Definition
Albergaria-Almeida (2010), Atwood and Stevens (1976), Bilaloğlu et al. (2017), Chewprecha et al. (1980), DeTure (1979), Gambrell (1983), Gorf and Roumagoux (1983), Hindman et al. (2019), Jegede and Olajide (1995), Murcia and Sheffield (2010), Patrick and Urhievwejire (2012), Rowe (1974), Riley (1986), Shrum (1984, 1985), Swift and Gooding (1983)	Wait Time 1: the pause after a teacher solicitation and before a student response or another teacher utterance Wait Time 2: the pause after a student response and before a teacher utterance
Heinze and Erhard (2006)	Wait Time 1: pause after a teacher question and before the end of a teacher utterance (i.e., in the case where the teacher continues talking after posing a question) Wait Time 2: pause after a teacher question and before a student response (i.e., the sum of pauses 1 and 3), Wait Time 3: the pause after the end of a teacher’s utterance (not question) and before a student’s response.
Dhindsa (2010)	Wait Time 1 is divided into: (1) Pause after a question addressed to the whole class (i.e., class answered in chorus), (2) Pause after a question addressed to an individual student and before the student answer (3) Pause after a question addressed to an individual student and before the teacher personally provided the answer or called on a different student.
Rezmer et al. (2020)	Wait Time 2 divided as: (1) Pause between the interviewer’s prompt and the child’s answer (2) Pause within the child’s narrative
Tobin (1980), K. Tobin (1984, 1986), K. G. Tobin and Capie (1982), Vettel and Windsor (1997)	Any pause preceding a teacher utterance
Baysen and Baysen (2010)	The length of pause preceding further speech by the same or different speaker
Lee et al. (1987)	Pause between the presentation of verbal stimuli and when the teacher poses a related question

### Natural Wait Time

In all, 23 studies measured adult’s wait time in naturalistic settings (i.e., without training or manipulation). In total, 19 studies adopted Rowe’s (1974) definition of wait time, among which all 19 recorded a measurement of Wait Time 1 and seven recorded a measurement of Wait Time 2. Of these articles, three reported instances of pause over a certain threshold, and as such, will not be included in the calculation of the average wait time (Albergaria-Almeida, 2010; Hindman et al., 2019; Murcia & Sheffield, 2010). Given Heinze and Erhard’s (2006) in-depth definitions of wait time, only Wait Time 3 (i.e., pause after a teacher finishes speaking and before a student responds) will be used in the calculation of average wait time, as it most closely resembles the measurement of other articles.

#### Wait Time 1

It was observed that adults’ natural Wait Time 1 ranged from 0.97 to 3.70 s, averaging **2.04 s** across studies. The shortest (under 1 s) was observed in elementary classrooms in the United States (DeTure, 1979; Gambrell, 1983) and the longest (over 3 s) was observed in elementary math classrooms in the United States and middle and high school science classrooms in Nigeria and Brunei (Dhindsa, 2010; Gorf & Roumagoux, 1983; Jegede & Olajide, 1995; Patrick & Urhievwejire, 2012). Of the three articles that reported pauses over a certain threshold, natural Wait Time 1 was short. In a lesson, Murcia and Sheffield (2010) identified only four instances of pause longer than 2 s, Hindman et al. (2019) identified two instances of pause longer than 1 s, and Albergaria-Almeida (2010) identified a mere 13% of questions that were given pauses longer than 2 s by teachers.

#### Wait Time 2

Adult’s natural Wait Time 2 was much shorter, ranging from 0.55 to 0.90 s and averaging ***0.69 s*** across seven studies. Similarly, <10% of questions were given pauses longer than 2 s (Albergaria-Almeida, 2010).

#### Wait Time General

The remaining four articles adopted Tobin’s definition of wait time, and as such, did not distinguish between Wait Time 1 and 2 (Tobin, 1980; K.Tobin, 1984, 1986; K. G.Tobin & Capie, 1982). Results indicated a natural wait time that ranged from 0.50 to 1.07 s, with an average of ***0.92 s***.

### Wait Time After Training

Nine studies measured wait time after adults received various types of training targeted to help increase classroom pauses. Training included pamphlets, audio and video tapes, interactive whiteboard tools, training groups, feedback devices (i.e., devices that prompt teachers to pause), and feedback groups (i.e., groups that provide teacher feedback and suggestions regarding their use of wait time before/after each lesson). All training targeted *all* wait times.

#### Wait Time 1

Six articles adopted Rowe’s (1974) definition of wait time, among which all reported a measurement of Wait Time 1. With training, teachers were able to increase their Wait Time 1 by 0.09 to 2.22 s, resulting in an average post-training Wait Time 1 of ***2.47 s*** (a range of 1.35–4.64 s). Two studies reported instances of pauses above a certain threshold, and both also found successful results (Albergaria-Almeida, 2010; Murcia & Sheffield, 2010). Murcia and Sheffield (2010) reported 28 more instances of Wait Time 1 exceeding 2 s, and Albergaria-Almeida (2010) observed a 25% rise in questions using Wait Time 1 over 2 s.

#### Wait Time 2

Four articles reported a measurement of Wait Time 2 under Rowe’s (1974) definition. The training was effective in extending Wait Time 2 as well, yielding an increase of 0.13 to 2.10 s and an average post-training pause of ***2.29 s*** (a range of 0.68–4.00 s). Albergaria-Almeida (2010) also observed an over 12% increase in questions using Wait Time 2 over 2 s. The most successful training to increase adult wait time includes pre-lesson training groups, feedback devices, and pamphlets.

#### Wait Time General

Three articles adopted Tobin’s definition of wait time. In comparison to the control group, teachers who received training demonstrated a 0.15 to 3.30 s increase in wait time and an average pause of ***3.11 s***. The most effective training was feedback groups for wait time and questioning practices. No studies examined the long-term effects of wait time training and whether extended wait time continues to be utilized in classrooms without training.

#### Effects of Extended Wait Time

The effects of extended wait time were reported in six studies, all of which took place in classrooms. Overall, when teachers extend both wait times beyond 3 s, significant changes were brought about in both student and teacher discourse. Not only did students engage more actively in class (i.e., increased student talk and questions, longer responses, and more idea exchange between peers), but they also exhibited enhanced critical thinking, offering higher quality utterances (i.e., increased relevance, incidence of speculative thinking, and provide more evidence in their responses). Consequently, students displayed more confidence, and these changes are reflected positively in their academic achievement and test scores. On the other hand, teachers speak and interrupt students less, instead, focusing on fostering student engagement and cultivating critical thinking by posing a greater variety of questions (i.e., more high-level and application questions, less rhetorical questions). Teachers further respond to students with more flexibility, soliciting and probing rather than mimicry and low-level reactions. A summary of the findings is shown in Table 2.

**Table 2.** Summary of Findings Related to Extended Wait Time by Themes.

Finding	K. Tobin (1984)	K. Tobin (1986)	DeTure (1979)	Rowe (1974)	Swift and Gooding (1983)	Tobin and Capie (1982)
Dec. overall utterance	X	X				
Student engagement						
Inc. student talk			X			
Inc. student response length	X	X		X		
Inc. idea exchange between students, student-to-student pairing			X	X		
More equal verbal contribution			X			
Inc. volunteered and relevant responses				X	X	
Inc. number of student questions				X		
Student critical thinking						
Inc. student speculative thinking				X		
More evidence in student response				X		
Other student behavior						
Inc. confidence				X		
Inc. contribution from students who do not usually contribute/slow				X		
Dec. failure to respond		X		X		
Higher mean scores/achievement	X	X			X	X
Teacher talk						
Dec. length/proportion of teacher talk	X	X	X		X	
Dec. teacher-centered inquiries			X			
Dec. teacher interruptions		X				
Inc. variety of questions				X		
Inc. evaluative, high level, application questions		X			X	
Dec. chain questions					X	
Teacher response						
Less mimicry/low-level utterance	X	X				
Inc. response flexibility				X		
Inc. proportion of soliciting, structuring, and probing	X	X				
Changed expectations toward students				X		

*Note*. Inc. = increase; Dec. = decrease.

One study identified a maximum threshold for classroom wait time. K. G.Tobin and Capie (1982) found that intermediate wait time (3 s) was associated with the highest attending rates when questions were highly relevant and clear.

### Wait Time Manipulated

Four studies experimentally manipulated adult wait times to compare the effects of brief (1 s) and extended (3–10 s) pauses. All articles found that extended wait time yielded positive results. Lee et al. (1987) examined the impact of teacher wait time on one-on-one instruction of imitation tasks with preschool children. The teachers were instructed to wait 1 s between presenting the stimuli and asking a question in the first trial, and 5 s in the second trial. Findings revealed that atypically developing children (i.e., Down syndrome, cerebral palsy) responded with higher frequencies, and as such, increased correct responses under 5 s wait time. No significant difference was found among typically developing children. To examine the effect of wait time on older children, Riley (1986) compared scores on a science test across classrooms taught by teachers instructed to maintain a wait time of 1, 3, or 5 s. Students taught by teachers with the longest wait time scored significantly higher on higher cognitive questions, but not on lower cognitive questions. It was found that as wait time increased from 3 to 5 s, students in the low-level group (i.e., teachers instructed to ask low-cognitive level questions) scored lower on lower cognitive questions. The author concluded that an upper threshold for wait time likely exists for lower cognitive questions only. Similarly, Tobin (1980) found that in comparison to the control group (i.e., under 1 s wait time), science achievement was significantly higher among students taught by teachers who maintained a 3 s wait time. However, this improvement only happened in the last phase of the 13-week period.

Finally, one study examined manipulated wait time in a forensic interviewing setting (Rezmer et al., 2020). Four- to eight-year-old children were interviewed about a science experiment they participated in approximately 6 days earlier with open-ended prompts (e.g., “tell me everything you can”). Interviewers were instructed to wait 10 s after a child stopped speaking before delivering the next prompt. First, it was found that the average time it took children to begin a productive response was ***4.80 s***. In fact, 46% of children had yet to respond to an open-ended prompt after a 5 s pause and 10% of children had yet to respond by the 10 s mark. Children took advantage of the silence they were provided even after they started their narrative. The average pause within a child’s narrative (i.e., the child begins talking, pauses, then continues talking) was ***6.60 s***. Although younger children usually continued their narrative after a 4 s pause, older children paused up to 10 s.

### Wait Time Across Question Type

While question type was commonly examined (*n* = 18), few articles compared wait time across different question types (*n* = 4). Three of four studies did not find a significant difference in teacher or adult wait time between question types (Atwood & Stevens, 1976; Heinze & Erhard, 2006; Vettel & Windsor, 1997). That is, regardless of what kind of question the teacher or adult is posing (e.g., open/closed), children were given the same amount of time to respond. Teachers in the remaining study gave significantly longer wait time for text-based questions (i.e., questions with obvious answers on the page; 1.06 s) than to script questions (i.e., questions which do not have obvious answers on the page and required using the readers’ script or experience, 0.78 s) in third-grade language art classrooms (Gambrell, 1983).

### Other Differences in Wait Time

Five articles compared wait time across ability groups. Two found that students expected to do well, or high performers, received significantly longer wait times from teachers compared to students not expected to do well (Bilaloğlu et al., 2017; Rowe, 1974), and another found significantly longer teacher wait time allocated to the highest and lowest performers compared to average performers (Shrum, 1984). The remaining two studies did not find a difference across ability groups (Gambrell, 1983; Gorf & Roumagoux, 1983). Several other variables were examined. Questions directed to boys (Gorf & Roumagoux, 1983), to individual students (Dhindsa, 2010), and questions asked in the native language in a second-language class (i.e., English questions in French class; Shrum, 1984) received significantly longer wait time (Table 3).

**Table 3.** Summary of Included Articles: Natural WT, Post-Training WT, and Manipulated WT in Seconds.

Study	Sample	WT1	WT2	Training/Manipulation	Main findings	Length	Quality
Albergaria-Almeida (2010) *Portugal*	Science classes (gr. 8), teachers (*N* = 3)	13% >2.00 *After training* 38% >2.00	<10% >2.00 *After training* 32% >2.00	Professional development course (2 months)	The course was successful in increasing both WT1 and WT2 to a length of over 2 s	5	12
Atwood and Stevens (1976) *U.S.*	Science classes (high school), teachers (*N* = 14)	1.01 Memory Qs: 0.95 High-cognitive Qs: 1.06	—	—	Students are well conditioned to respond to questions quickly. WT1 did not differ depending on the level of questions the teacher asked	6	15
Baysen and Baysen (2010) *Turkey*	Science classes (elementary), perspective teachers (*N* = 35)	2.36	—	—	WT may be culturally dependent.	5	13
Bilaloğlu et al. (2017) *Turkey*	Science classes (preschool, 6-year-olds), teachers (*N* = 6).	1.15	0.58	—	Teachers did not meet wait time guidelines. Students expected to do well-received sig. longer time.	17	19
Chewprecha et al. (1980) *Thailand*	Science classes (high school), teachers (*N* = 77)	2.42 *After training* G1: 4.64 G2: 2.92 G3: 2.51	—	G1: Pamphlet G2: Qualitative audio tape G3: Quantitative audio clip	Pamphlets on questioning techniques and WT guidelines are significantly more effective than audio clip training	10	14
DeTure (1979) *U.S.*	Classrooms (gr. 4–5), preservice teachers (*N* = 52)	0.99 *After training* G1: 0.99 (+0.00) G2: 1.52 (+0.53) G3: 1.23 (+0.24) G4: 1.06 (+0.07)	0.60 *After training* G1: 1.50 (+0.90) G2: 1.81 (+1.21) G3: 2.09 (+1.49) G4: 2.29 (+1.63)	G1: Audio without feedback G2: Audio with feedback G3: Video without feedback G4: Video with feedback	Video and audio modeling are successful tools, but not to the point of ideal WT. Video modeling is more effective. Extended WT decreased teacher-centered inquiry patterns.	10	14
Dhindsa (2010) *Brunei*	Science classes (high school), teachers (*N* = 15)	Whole class: 3.70 Individual: 4.20 Teacher intended: 5.60	—	—	WT differs depending on who the question was addressed to	16	18
Gambrell (1983) *U.S.*	Language Arts classes (gr. 3), teachers (*N* = 9)	0.97 Text-based Qs: 1.06 Scriptal Qs: 0.78	—	—	WT does not differ across ability groups, but sig. different across question types.	5	15
Gorf and Roumagoux (1983) *U.S.*	Math classes (gr. 4), teachers (*N* = 5)	*Gender: 3.25* Girls: 2.80 Boys: 3.70 *Achievement: 3.39* Low: 3.67 Med: 3.41 High: 3.09	—	—	WT not sig. different across achievement groups. Sig. longer WT for boys than girls.	3	14
Heinze and Erhard (2006) *Germany*	Math classes (gr. 8), teachers (*N* = 22)	WT1^a^: 5.60 WT2^a^: 4.20 WT3^a^: 2.50	—	—	75% of WT3 is under 3 s. Teachers continued talking after asking a question in 1/3 of cases.	11	18
Hindman et al. (2019) *U.S.*	Language Arts classes (3–4-year-olds, 10% with special needs), teachers (*N* = 27)	2 instances of <1.00	—	—	Two teachers provided WT around 1 s. WT was not observed in other teachers. Rapid fire approach observed.	18	19
Jegede and Olajide (1995) *Nigeria*	Science classes (gr. 8–10), teachers (*N* = 27)	3.00	0.74	—	Classrooms with long WT are associated with more student attempts and more time teachers spend questioning. Classrooms with short WT are associated with less time teachers spend questioning.	17	17
Lee et al. (1987) *U.S.*	Home (24–34-months-old), teachers (atypically developing *n* = 2, TD *n* = 1)	—	—	WT1: Trial 1: 1.00 Trial 2: 5.00	For atypically developing children only, longer WT is associated with higher response frequencies and increased overall correct responses.	9	16
Murcia and Sheffield (2010) *Australia*	Science classes (10–12-year-olds), teachers (*N* = 4)	4 instances of >2.00 *After training*: 30 instances of >2.00	—	Interactive whiteboard tools	The interactive whiteboard is successful in helping teachers increase WT1.	15	11
Patrick and Urhievwejire (2012) *Nigeria*	Science classes (high school), teachers (*N* = 60)	3.10	—	—	Most questions asked received 3 s WT.	9	18
Rezmer et al. (2020) *U.S.*	Laboratory (4–8-year-olds *N* = 105; *M*_age_ = 6.14), interviewers.	—	—	WT1&2: 11.95	Interviewers were able to follow 10 s WT but found it unnatural. 46% of children did not respond by 5 s, 10% did not respond by 10 s. Younger children paused up to 4 s within their narrative, older children paused up to 10 s	9	20
Riley (1986) *U.S.*	Science classes (gr. 2–5), preservice teachers (*N* = 26)	—	—	WT1 G1: 1.00 G2: 3.00 G3: 5.00	Students taught by teachers with 5 s WT scored significantly higher on comprehension, but not on knowledge questions.	8	16
Rowe (1974) *U.S.*	Science classes (gr. 1–6), teachers (*N* = 12)	1.00 *After training* 3.00 (+2.00)	0.90 *After training* 3.00 (+2.10)	Training groups	The training was successful in increasing both WTs to 3 s. Teachers upheld this in classrooms.	22	16
Shrum (1984) *U.S.*	Second language classes (first-year high school, *N* = 5 classes), teachers	1.91 Performance High: 2.69 Ave: 1.87 Low: 2.18	0.73 Performance High: 0.83 Ave: 0.74 Low: 0.56	—	WT1 sig. longer for high and low performers than for average performers. WT was observed after 94% of solicitations and after 90% of student responses.	7	18
Shrum (1985) *U.S.*	Second language classes (first-year high school, *N* = 5 classes), teachers	1.91 English Qs: 2.33 Target language Qs: 1.70	0.73 English Qs: 0.70 Target language Qs: 1.11	—	WT1 after English solicitations were sig. longer than after target language solicitations.	10	18
Swift and Gooding (1983) *U.S.*	Science classes (middle school), teachers (*N* = 40)	1.18 *After training* G1: 1.19 (+0.01) G2: 1.35 (+0.17) G3: 2.62 (+1.44) G4: 1.80 (+0.62)	0.55 *After training* G1: 0.54 (−0.01) G2: 0.68 (+0.13) G3: 1.36 (+0.81) G4: 0.97 (+0.42)	G1: Control G2: Printed guides G3: Feedback devices G4: Guides and feedback devices	Feedback training was successful in sig. increasing WT, however, WT1 still did not reach the 3 s mark. Benefits to teacher and student discourses brought about by feedback and guides group.	10	17
Tobin (1980) *Australia*	Science classes (10–13-year-olds), teachers (*N* = 23)	0.50	—	G1 control: 0.70 G2 manipulated: 3.10	WT sig. correlated with science achievement on two tests after manipulation, but not before.	7	16
K. Tobin (1984) *Australia*	Classrooms (gr. 6–7), teachers (*N* = 20)	0.90 *After training* 3.30 (+2.40)	—	Feedback groups	Feedback groups successfully increased teacher WT to meet 3–5 s guideline. Benefits observed.	13	19
K. Tobin (1986) *Australia*	Math and Language Arts classes (gr. 6–7), teachers (*N* = 20)	Math: 0.90 L.A.: 1.50 *After training* Math: 3.30 (+2.10) L.A.: 4.50 (+3.30)	—	Feedback groups	Feedback groups successfully increased teacher WT to meet 3–5 s guideline. Benefits observed.	10	17
K. G. Tobin and Capie (1982) *Australia*	Science classes (gr. 6–8, *N* = 13 classes), teachers	1.07 *After training* G1: 2.35 (+1.28) G2: 1.22 (+0.15) G3: 3.97 (+2.90)	—	G1: WT feedback groups G2: Questioning feedback groups G3: WT & Questioning feedback groups	WT feedback groups were successful. Sig. interaction between WT, cognitive level of questioning, questioning relevance, and clarity	14	19
Vettel and Windsor (1997) *U.S.*	Laboratory, mothers. DS (*N* = 8; *M*_age_ = 4;4) TD (*N* = 8; *M*_age_ = 1;9)	DS: 1.80 TD 2.50	—	—	Mothers of children with DS provide sig. shorter WT1. Mothers did not differentiate between question types.	8	6

*Note*. WT = wait time; gr. = grade; s = seconds; G = group; Qs = questions; Ave = average; TD = typically developing; DS = Down syndrome.

a See text for Heinze and Erhard’s (2006) definition of the three types of wait time.

## Discussion

The present systematic review synthesized existing literature across various fields regarding the role of wait time when questioning children. Several insights into the significance of wait time in adult-child discourse and the effects of extended wait time were identified. Most research on wait time has stayed within the field of education. Only one study in the current review examined wait time in a forensic interviewing setting (Rezmer et al., 2020). Despite this, the findings provide insight into how pauses may be used to support children when responding to adults’ questions, such as during forensic interviews. Most studies in the current review measured and examined wait time using Rowe’s (1974) definition, dividing pauses between Wait Time 1 and Wait Time 2, with a handful adopting Tobin’s (1980) broader definition.

The current review found that the natural wait time adults give for children to answer was short, but variable. After hearing a question, children may be given as little as under 1 s to as much as nearly 4 s to provide an answer. Interestingly, while one may expect that the difficulty of the question being asked may predict the amount of wait time allotted, adult’s wait time did not reliably differ across question types, and when it did, it differed in a counter-intuitive way. Adults’ wait times across question types were compared in four studies and revealed that they give children the same (Atwood & Stevens, 1976; Heinze & Erhard, 2006; Vettel & Windsor, 1997) or less amount of time (Gambrell, 1983) to answer higher cognitive level questions (e.g., open-ended questions). These results contrast the findings that when given as much time as needed, higher cognitive level questions take significantly longer to answer for both college (Ellsworth et al., 1991) and elementary school students (Arnold et al., 1974). Forensic interviewers are encouraged to use open-ended, high cognitive-level questions. If the patterns observed in classrooms are also present in forensic interviewing, these findings could suggest that child witnesses are not getting enough space to answer the most difficult questions. Future research must examine if such a pattern exists in a forensic setting. Furthermore, the current review did not examine whether wait time should vary across cognitive levels and age. Provided that questions posed to children at different levels may vary in difficulty both in forensic interviewing and in classrooms, future research should also investigate whether wait time allocated to children at different ages should vary as well.

One factor that was reliably related to variations in adults’ natural wait time was whether questions were asked in a multilingual setting. English classrooms in countries where English is not the primary language observed some of the longest wait times (Dhindsa, 2010; Gorf & Roumagoux, 1983; Jegede & Olajide, 1995; Patrick & Urhievwejire, 2012). A possible explanation is that these teachers were aware that students may need additional time to translate English instructions to their native language, and then provide a response in English. This was not observed in second-language classes in the United States, where teachers provided shorter wait times for questions asked in the target language than questions asked in English (Shrum, 1984). This is an important area of future research for considering wait times in the legal context because forensic interviewers must also recognize that child witnesses interviewed in a second language may require more time to respond. While current interviewing protocols recommend clearer and slower enunciation of questions (e.g., Lamb et al., 2018) when speaking to non-native English speakers, it is important, especially for English-speaking forensic interviewers, to provide sufficient wait time. How culture and language affect the natural use of wait time with children must continue to be examined in forensic interviewing among diverse samples.

Regarding experimentally manipulated wait time, the current review indicated a robust finding of extended wait time consistently yielding positive outcomes across settings (i.e., classrooms, labs, homes). Several trainings have been identified to effectively extend adult wait time, including pre-lesson training groups and feedback groups. Moreover, it was observed that a teacher wait time beyond 3 s brought about notable effects in the classroom. Wait time within this range was associated with, but not limited to, teachers asking more diverse and high-level questions and students responding with more confidence. The noted improvements are equally beneficial for forensic interviews and court testimonies. As mentioned, posing higher-level, open-ended questions is critical in forensic interviews as they can elicit longer answers compared to close-ended questions (Brubacher et al., 2019; Lamb et al., 2018). Furthermore, reducing “I don’t know” responses with the wait time (Rowe, 1974) could be especially important for reluctant child witnesses who tend to use such responses to avoid providing an answer (Ahern et al., 2019; Hershkowitz et al., 2006). Beyond forensic interviewing, the extended wait time could be relevant when children are testifying in the courtroom. Canadian courts, for example, do not currently have regulations governing how long one must wait for a child witness to respond before considering them unresponsive. Testifying and undergoing cross-examination can be especially stressful for children who have experienced maltreatment. While these factors are considered regarding unresponsive witnesses (R. v. Hart, 1999; R. v. T.H., 2017) and judges are encouraged to do what they can to reduce unresponsiveness (R. v. L. (D.O.), 1993), Canadian law does not specify how *long* a witness must remain silent to be considered unresponsive. In R. v. Hart (1999), for instance, a 12-year-old witness testifying for two alleged sexual assaults was identified as unresponsive. However, it was not indicated how long the defense counsel waited for the child to answer before following up with further questions. Should the child have required more time to process the question or the emotions behind testifying, it is likely they were not provided this time. This could, in turn, largely alter the decision of the case.

Conversely, one study noted that the attending rate (i.e., looking at the teacher, worksheet, etc.) dropped when the wait time was beyond 3 s ( K. G.Tobin & Capie, 1982). Another found that student achievement on lower-level cognitive questions dropped when teacher wait time went from 3 to 5 s (Riley, 1986). Researchers have therefore encouraged teachers to maintain a wait time of 3 to 5 s to allow for cognitive processing and high-quality classroom discourse. This could be achieved with low-cost training like pamphlets and feedback.

Only two studies in the current review examined and reported negative effects of extended wait time, and none examined what happens to discourse when wait time is extended beyond 5 s. Indeed, K.Tobin’s (1987) review also cautioned that while findings of extended wait time are promising, the silence used to encourage cognitive processing may be less useful during simple or repetitive instructions (e.g., rote learning). Despite this, research in education has yet to explore whether a maximum threshold for teacher wait time is needed. Long-term effects of wait time training (e.g., whether teachers uphold extended wait time) were not examined. When developing a wait time guideline for forensic interviewing, such limitations must be considered. For instance, a maximum wait time to prevent losing children’s focus and using shorter pauses when asking close-ended questions may be more productive in interviews.

The role of wait time in a forensic interviewing setting was examined in one study (Rezmer et al., 2020). Interviewers in the study were successful in waiting at least 10 s before and after children’s responses. Contrary to findings from classrooms, it was observed that on average, children needed over 6 s to respond to an open-ended prompt to recall a past event. Even within their narrative, older children may pause up to 10 s before continuing. Should the interviewers have followed the 3 to 5-s wait time guideline from education, many children would have been cut off before they could answer or during their response. Given that 96% of productive pauses following an open-ended prompt and that 97% of productive pauses within a child’s narrative occurred at or before 10 s, Rezmer et al. (2020) concluded that, unlike classrooms, a 10-s guideline is more appropriate when conducting forensic interviews with children. One possibility is that the setting of a forensic interview greatly contrasts that of a classroom discussion. First, forensic interviews are mostly conducted in a one-on-one setting, which children may interpret as a less competitive space and allow themselves more time to think before answering. The demands also differ greatly across these settings. In classrooms, children are tasked to learn (e.g., store information in long-term memory and apply information to questions) and are asked about learning materials or personal experiences. In forensic interviews, however, they are tasked to recall and describe, in detail, an event that happened in the past (i.e., retrieve information from memory). The long pause within the children’s narrative documented in Rezmer et al.’s (2020) study demonstrates that they took advantage of the space provided to continue recalling details of the event as they were responding. Lastly, events children are tasked to recall in the legal context tend to be emotional and sensitive (e.g., accounts of abuse). The emotionality and other concerns children may have regarding such disclosures (e.g., wanting to protect a familiar perpetrator; Paine & Hansen, 2002) all contribute to potentially requiring more time. Provided how varied the dynamics and demands are within these two settings, adults must also wait differently. There is a dire need for more research examining the role of wait time in the legal context and the unique factors that exist in this setting. Although interviewers in Rezmer et al.’s (2020) article were successful in maintaining a 10 s wait time, the effect of wait time training in education has yet to be explored for forensic interviewers. Before that, we must be cautioned when applying what was found in classrooms directly to forensic interviews.

### Implications for Forensic Interviewing and Directions for Future Research

In light of the findings, several implications for child forensic interviewing arise. First, across studies that examined the effect of extending wait time beyond 1 to 2 s, we found a consistent, positive effect on adult–child discourse across multiple settings. These findings suggest that extended wait time could be adopted by child forensic interviewers, and may be a simple way to elicit longer, more confident, and speculated responses, even from children who usually do not feel comfortable participating. Integrating wait time into forensic interviews with child witnesses will be a critical next step for interview protocols and practices. However, we only identified one study in the current review which examined the role of wait time in an interviewing context. A key direction for future research is to continue determining how findings on wait time in education could be translated to psychology and forensic psychology. Future research should explore topics including current practice and perception of wait time among child forensic interviewers, how varying wait time lengths affect the productivity of children’s testimonies and disclosures, and whether wait time beyond 10-s length brings about a negative impact in the legal context. The design of future studies should also investigate whether wait time should differ across various question types (i.e., open- and close-ended) and age, and its utility among reluctant or non-disclosing child witnesses.

A second implication relates to the finding that while natural wait time is very short, several trainings have been demonstrated to be effective in extending adult pauses. Many trainings identified in the current review are low-cost and simple (e.g., pamphlets, receiving feedback), meaning that not only forensic interviewers, but other common adult recipients of maltreatment disclosures (e.g., teachers, police officers, social workers, parents) could benefit from wait time training to help elicit as detailed of disclosure from children as possible. Future research should explore what training is currently used or available for forensic interviewers, if any, and to develop evidence-based wait time training for forensic interviewers and other professionals, drawing upon knowledge and practices established in the field of education (Table 4).

**Table 4.** Implications of the Review.

Area of Focus	Implication
Practice	Extended wait time should be adopted by child forensic interviewers. May be a way to elicit higher-quality responses, even from reluctant children. Low-cost training on wait time should be implemented for forensic interviews and other common adult recipients of maltreatment disclosures.
Policy	Development of evidence-based guidelines and training on wait time in forensic and court interviewing must begin by drawing upon knowledge established in other fields.
Research	Research is needed on wait time in the context of forensic interviewing and courtroom testimony. Topics to explore include how various lengths of wait time affect the productivity of testimonies and disclosures, the effects of wait time beyond 10 s, the utility of wait time across question types and reluctant children, and training on wait time in forensic interviewing.

### Limitations

Several limitations must be considered when interpreting the results of the current study. First, only studies published in English were included in the current review, which may have excluded significant research conducted in other languages. Given the potential difference in conversation and pauses across languages, findings may differ when examining the questioning of children in a different language. Second, in contrast to the previous wait time review ( K.Tobin, 1987), we did not include gray literature and unpublished dissertations. How wait time varies across ages was also not examined in this review. Finally, we also acknowledge that given the extent of published and unpublished studies worldwide, the search was restricted to specific search engines, journals, and search terms. The authors also recognize that the COVID-19 pandemic has impacted researchers and brought delays to the academic community worldwide. This review was conducted at a time when the world is just coming out of the pandemic, hence studies whose publication was delayed due to the pandemic may not have been included.

## Conclusion

The current systematic review aimed to provide a holistic update on the role of wait time in questioning children to determine whether this could be an interviewing practice that is effective in forensic interviews. Overall, results indicated that a wait time of 3 to 5 s had a significant impact on classroom discourse that should be recognized by researchers in forensic psychology. In the legal context, however, a wait time of 10 s may be more suitable. Practically, these findings should act as a call for research to further examine how wait time can be used as an effective interviewing practice for facilitating children’s disclosure. This will be crucial to begin informing best-practice wait time guidelines in the legal context.

## Supplemental Material

sj-docx-1-tva-10.1177_15248380241246793 – Supplemental material for The Role of Wait Time During the Questioning of Children: A Systematic Review Supplemental material, sj-docx-1-tva-10.1177_15248380241246793 for The Role of Wait Time During the Questioning of Children: A Systematic Review by Annie Yun An Shiau, Kelly McWilliams and Shanna Williams in Trauma, Violence, & Abuse
