# Supplementary figures and images for: The Role of Wait Time During the Questioning of Children: A Systematic Review
Source: Trauma Violence Abuse. 2024 Apr 25;25(5):3441–56. doi: 10.1177/15248380241246793 (PMC11545128; doi:10.1177/15248380241246793)

**Appendix A.**

*Visual Representation of Common Wait Time Definitions.*


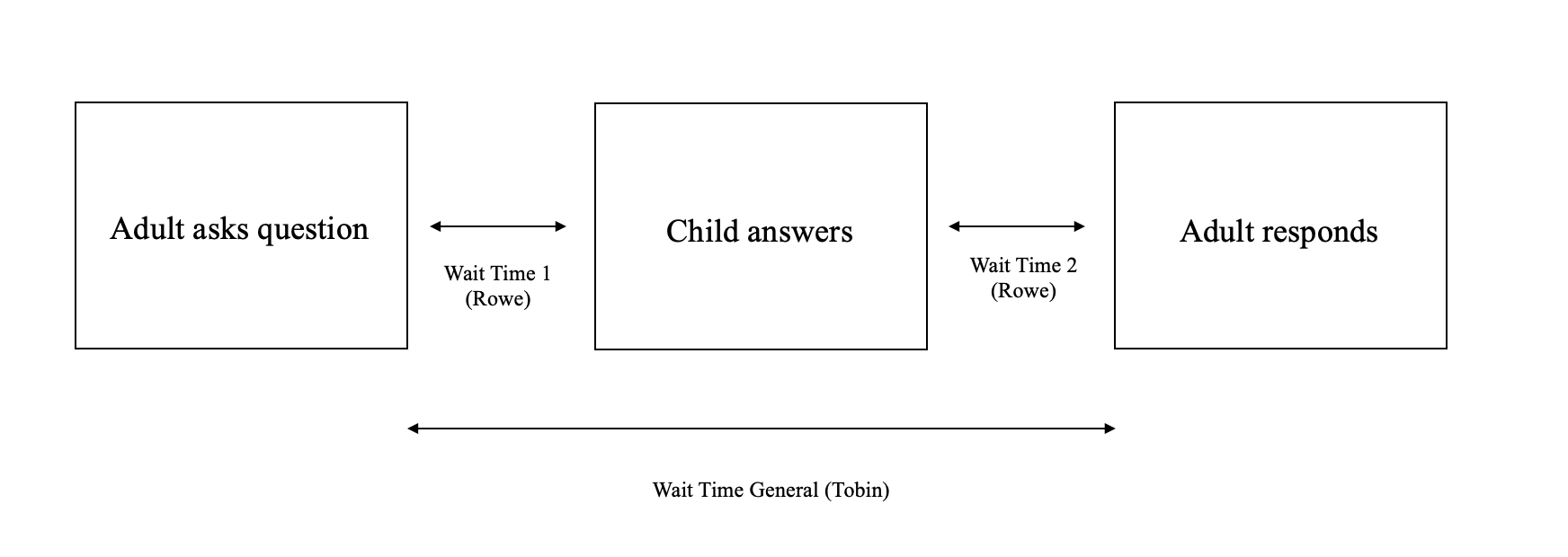

Supplement: sj-docx-1-tva-10.1177_15248380241246793 – Supplemental material for The Role of Wait Time During the Questioning of Children: A Systematic Review [file sj-docx-1-tva-10.1177_15248380241246793.docx]
